# Heat-Shock Proteins in Autoimmunity

**DOI:** 10.1155/2013/621417

**Published:** 2013-05-19

**Authors:** Kamal D. Moudgil, Stephen J. Thompson, Fabiana Geraci, Boel De Paepe, Yehuda Shoenfeld

**Affiliations:** ^1^Department of Microbiology and Immunology, University of Maryland School of Medicine, Baltimore, MD 21201, USA; ^2^Department of Academic Rheumatology, King's College, London SE1 1UL, UK; ^3^Department of Cellular and Developmental Biology (STEMBIO), University of Palermo, Viale delle Scienze, 90128 Palermo, Italy; ^4^Department of Neurology and Neuromuscular Reference Center, Ghent University Hospital, 9000 Ghent, Belgium; ^5^Zabludowicz Center for Autoimmune Diseases, Sheba Medical Center (Affiliated to Sackler Faculty of Medicine, Tel-Aviv University), 52621 Tel-Hashomer, Israel

Heat shock proteins (HSPs), also known as “stress proteins,” are among the highly conserved and immunogenic proteins shared among diverse groups of microbial agents and mammals [1]. Heat and other types of stressful stimuli can increase the cellular expression of HSPs. These proteins have been categorized into different families according to their molecular mass, for example, HSP110, HSP90, HSP70, HSP60, HSP40, HSP20-30, and HSP10 [1–3]. For uniformity, guidelines for the nomenclature of various human HSP families have been proposed [4]. Under physiological conditions, the ubiquitously distributed HSPs maintain the integrity and function of other cellular proteins in stressful conditions. However, HSPs also can become targets of immune response, resulting in immune pathology and clinical manifestations of autoimmunity. HSPs have been implicated in the pathogenesis of a variety of autoimmune diseases [3, 5–7]. Moreover, the involvement of other small HSPs (e.g., H11/HspB8) besides the canonical members of that family in health and disease is increasingly being realized. Importantly, HSPs are also capable of inducing immune responses that are immunoregulatory in nature [7–10], including resolution of the inflammatory responses [11]. Also, the concept of immune network (immunological homunculus) including HSPs as one of its components has been proposed to explain immune homeostasis in health and disease [12]. 

The main focus of this special issue is on the role of different HSPs in the pathogenesis of autoimmunity through induction/propagation as well as regulation of the disease-related processes; on the impact of physiological and disease-related metabolic processes on the induction of HSPs; on the mechanistic basis of the effector functions driven by HSPs; and on the immunomodulatory role of HSPs. Thirteen excellent papers describing new original results and the most recent developments in the field on these topics are presented in this special issue. These papers cover the role of HSPs in antigen cross-presentation, induction of autoimmunity, and immunotherapy of autoimmunity/cancer (R. Binder et al., Y. Kato et al., and S. Calderwood et al.); the role of HSPs in the pathogenesis of the autoimmune components of diverse diseases including atherosclerosis (A. Kilic and K. Mandal); systemic lupus erythematosus (H. Shukla and P. Pitha); Behcet's disease (J. Shimizu et al.), uveitis (A. Commodaro et al.), and diabetes (C. Blasi et al.); the relationship between infection, particularly *M. paratuberculosis*, and autoimmunity (C. Dow); the role of small heat-shock protein H11/HspB8 and its homologous proteins in human disease (L. Aurelian et al.); the impact of exercise and metabolic disorders on HSPs (E. Noble and G. Shen); the immunosuppressive activity of HSP70 (P. Stocki and A. Dickinson); and HSP-induced regulatory T cells and their role in control of autoimmunity (E. Brenu et al.). These papers highlight results obtained from studies in animal models as well as patients with autoimmune or metabolic disorders.

HSPs are highly conserved in nature, and they are also quite immunogenic. These attributes may render these proteins as initiators of immune response as well as targets of autoimmune attack. Foreign (e.g., microbial) HSPs may prime cellular or humoral immune responses that might be cross-reactive with the corresponding self-HSPs or other self-antigens leading to the induction of autoimmunity. “Molecular mimicry” and unveiling of the previously cryptic (hidden) determinants are among the different mechanisms involved in the induction of autoimmunity [3]. Interestingly, foreign-self mimicry may not always be pathogenic; instead, it may be immunoregulatory in nature [13, 14]. Further, defined pathogenic versus regulatory T- or B-cell epitopes have been identified within the same HSP, for example HSP60/65. Accordingly, during the course of autoimmunity, epitope spreading or diversification of response can lead to induction of new T-cell/antibody responses, which in turn may aggravate or downmodulate autoimmunity depending on the antigenic epitopes targeted in the process. Several papers in this special issue discuss the pathogenic role of HSP-induced immunity. The role of particular HSPs in specific autoimmune disorders is highlighted in the papers on atherosclerosis (A. Kilic and K. Mandal), uveitis (A. Commodaro), lupus (H. Shukla and P. Pitha), and Behcet's disease (J. Shimizu et al.). A viewpoint on the role of *M. paratuberculosis* HSP65 in the induction of autoimmunity via molecular mimicry is presented by C. Dow. Two papers in this issue have addressed the relationship between HSPs and metabolic disorders with components of autoimmunity (E. Noble and G. Shen; C. Blasi et al.).

HSPs are known to play critical roles in innate immunity as well as adaptive immunity. HSPs can activate specific toll-like receptors (TLRs) and influence activation of antigen-presenting cells (APC) and thereby other immune cells including T cells and B cells. These proteins also play a major role in cross-presentation of extracellular antigens such as microbial antigens or tumor antigens in parenchymal cells via the class I pathway resulting in induction of CD8+ cytotoxic T-lymphocyte (CTL) responses [15, 16]. Cross-presentation of HSP-mediated presentation of self-antigens might also play a role in the pathogenesis of autoimmunity. The mechanistic basis of the role of HSPs in cross-presentation of antigens and the impact of this process on immune response in cancer and autoimmunity are elaborated in this issue in papers by S. Calderwood et al., R. Binder et al., and Y. Kato et al.

HSPs are induced by a variety of stressful stimuli, and they aid in controlling the physical and metabolic integrity of the cells under stress. Metabolic disorders and exercise can induce HSPs and activate other heat-shock factor-1- (HSF-1-) mediated effector pathways. These in turn can enhance the generation of mediators of inflammation. E. Noble and G. Shen discuss in this issue the above-mentioned associations, including their dual role in inflammation. However, C. Blasi et al. report that an effective control of type 2 diabetes was not accompanied by a reduction in serum levels of HSP60 and antibodies to HSP60, while it lowered the levels of the proinflammatory cytokine IL-6.

As elaborated above, HSPs are involved in the induction as well as regulation of immune responses. How the same proteins can mediate opposite outcomes is a dilemma for both basic researchers and physician investigators. This dual role of HSPs has been revealed in a wide variety of disorders, including autoimmune diseases and tumors, as well as in immune responses associated with organ transplantation [7, 8, 10, 13, 15, 17, 18]. It is becoming clear that the pro- versus anti-inflammatory activities of HSPs are contextual and affected by multiple factors including the concentration of HSP, the timing of exposure to HSP, and the overall physiopathological milieu at the target site. Another challenge regarding HSPs and immune responses lies in the fact that autoimmunity and tumors present with opposite requirements for control of the disease processes [2, 17]. Autoimmunity involves a breakdown of self-tolerance and induction of anti-self immune responses. Accordingly, an effective control of autoimmunity requires suppression of autoreactive immune responses. On the contrary, tumors survive and grow in the body owing to an ineffective antitumor response in part because of immune-evasive strategies adopted by tumor cells. A deliberate induction of potent antitumor immunity is required to control cancer. HSPs are being exploited differentially to control autoimmunity and cancer. The disparate roles of HSPs in tumor immunity and autoimmunity and the molecular and cellular mechanisms involved therein are discussed here by S. Calderwood et al., R. Binder et al., and L. Aurelian et al.

HSPs may facilitate regulation of effector responses under appropriate conditions [3, 7–9]. This can be achieved in part via increasing the production of anti-inflammatory cytokines so as to deviate the cytokine balance from a proinflammatory to an anti-inflammatory type. In addition, HSPs may induce different types of regulatory T cells, including CD4+ Tr1 cells that secrete interleukin-10 (IL-10) and CD4+CD25+ Forkhead-box-P3- (Foxp3-) expressing Treg that produce transforming growth factor-b (TGF-*β*) and IL-10. Treg may also mediate their effect via cell-to-cell contact with the target cells. In this issue, the immunosuppressive activity of HSP is outlined by P. Stocki and A. Dickinson, while HSP-induced regulatory cells are described by E. Brenu et al.

HSPs are being exploited for immunotherapy of autoimmune diseases and cancer [2, 15, 19, 20]. The current approaches under development or those being tested in clinical trials harness the immunoregulatory properties of HSPs. For example, purified HSPs or their peptides containing defined epitopes are being tested in clinical trials in type 1 diabetes [19] and rheumatoid arthritis [20] patients with the hope of developing immunotherapeutic approaches for these debilitating diseases. The use of HSP-induced regulatory T cells is an emerging area of potential promise in this regard [9]. 

We hope you enjoy reading the diverse collection of outstanding papers in this special issue.

## References

[1] Lindquist S, Craig EA (1988). The heat-shock proteins. *Annual Review of Genetics*.

[2] Srivastava P (2002). Interaction of heat shock proteins with peptides and antigen presenting cells: chaperoning of the innate and adaptive immune responses. *Annual Review of Immunology*.

[3] Rajaiah R, Moudgil KD (2009). Heat-shock proteins can promote as well as regulate autoimmunity. *Autoimmunity Reviews*.

[4] Kampinga HH, Hageman J, Vos MJ (2009). Guidelines for the nomenclature of the human heat shock proteins. *Cell Stress and Chaperones*.

[5] Shoenfeld Y, Harats D, George J (2000). Heat shock protein 60/65, *β*2-glycoprotein I and oxidized LDL as players in murine atherosclerosis. *Journal of Autoimmunity*.

[6] De Paepe B, De Bleecker JL (2013). The nonnecrotic invaded muscle fibers of polymyositis and sporadic inclusion body myositis: on the interplay of chemokines and stress proteins. *Neuroscience Letters*.

[7] Geraci F, Turturici G, Sconzo G (2011). Hsp70 and its molecular role in nervous system diseases. *Biochemistry Research International*.

[8] Harats D, Yacov N, Gilburd B, Shoenfeld Y, George J (2002). Oral tolerance with heat shock protein 65 attenuates Mycobacterium tuberculosis-induced and high-fat-diet-driven atherosclerotic lesions. *Journal of the American College of Cardiology*.

[9] van Herwijnen MJ, Wieten L, van der Zee R (2012). Regulatory T cells that recognize a ubiquitous stress-inducible self-antigen are long-lived suppressors of autoimmune arthritis. *Proceedings of the National Academy of Sciences of the United States of America*.

[10] Motta A, Schmitz C, Rodrigues L (2007). Mycobacterium tuberculosis heat-shock protein 70 impairs maturation of dendritic cells from bone marrow precursors, induces interleukin-10 production and inhibits T-cell proliferation in vitro. *Immunology*.

[11] Shields AM, Thompson SJ, Panayi GS, Corrigall VM (2012). Pro-resolution immunological networks: binding immunoglobulin protein and other resolution-associated molecular patterns. *Rheumatology*.

[12] Cohen IR (1992). The cognitive paradigm and the immunological homunculus. *Immunology Today*.

[13] Anderton SM, van der Zee R, Prakken B, Noordzij A, van Eden W (1995). Activation of T cells recognizing self 60-kD heat shock protein can protect against experimental arthritis. *Journal of Experimental Medicine*.

[14] Moudgil KD, Chang TT, Eradat H (1997). Diversification of T cell responses to carboxy-terminal determinants within the 65-kD heat-shock protein is involved in regulation of autoimmune arthritis. *Journal of Experimental Medicine*.

[15] Calderwood SK, Ciocca DR (2008). Heat shock proteins: stress proteins with Janus-like properties in cancer. *International Journal of Hyperthermia*.

[16] Binder RJ, Srivastava PK (2005). Peptides chaperoned by heat-shock proteins are a necessary and sufficient source of antigen in the cross-priming of CD8+ T cells. *Nature Immunology*.

[17] Coelho V, Broere F, Binder RJ, Shoenfeld Y, Moudgil KD (2008). Heat-shock proteins: inflammatory versus regulatory attributes. *Cell Stress and Chaperones*.

[18] Slack LK, Muthana M, Hopkinson K (2007). Administration of the stress protein gp96 prolongs rat cardiac allograft survival, modifies rejection-associated inflammatory events, and induces a state of peripheral T-cell hyporesponsiveness. *Cell Stress and Chaperones*.

[19] Huurman VAL, van der Meide PE, Duinkerken G (2008). Immunological efficacy of heat shock protein 60 peptide DiaPep277 therapy in clinical type I diabetes. *Clinical and Experimental Immunology*.

[20] Koffeman EC, Genovese M, Amox D (2009). Epitope-specific immunotherapy of rheumatoid arthritis: clinical responsiveness occurs with immune deviation and relies on the expression of a cluster of molecules associated with T cell tolerance in a double-blind, placebo-controlled, pilot phase II trial. *Arthritis and Rheumatism*.

